# Genetic variation in circadian regulator gene BMAL1 in psychiatric, psychological and cardiometabolic traits: a trans-ancestry UK Biobank study

**DOI:** 10.1136/bmjment-2024-301267

**Published:** 2024-12-12

**Authors:** Hamza Daudali, Jana Anderson, Mark E S Bailey, Alexander Fradera, Claire L Niedzwiedz, Donald Lyall, Laura M M Lyall, Rona J Strawbridge

**Affiliations:** 1 University of Glasgow, Glasgow, UK; 2 Department of Medicine, Karolinska Institute, Stockholm, Sweden

**Keywords:** PSYCHIATRY, Cross-Sectional Studies, Data Interpretation, Statistical

## Abstract

**Background:**

The link between cardiometabolic disease and mental illness has been well established but remains incompletely explained. One hypothesis suggests that circadian rhythm dysregulation links cardiometabolic disease and mental illnesses. *BMAL1* is a circadian rhythm regulatory gene. Human genetic studies have implicated *BMAL1* in depression, schizophrenia, bipolar disorder as well as body mass index, blood pressure and lipid levels.

**Objective:**

We investigated the *BMAL1* locus genetic variants for associations with both cardiometabolic and mental illness.

**Methods:**

Genetic and phenotypic data from UK Biobank (~500 000 participants) of White British, African-Caribbean, South Asian, white European and Multiple ancestries were used. Regression analyses using Plink 1.09 was used to identify significant associations, with Bonferroni multiple testing correction. Multiple ancestry meta-analyses using METAL software was used to investigate trans-ancestry consistency in genetic effects.

**Findings:**

We identified associations for body mass index, anhedonia, diastolic and systolic blood pressure, waist-hip ratio, major depressive disorder, neuroticism and risk-taking. Meta-analyses indicated that there are ancestry-wide and ancestry-specific effects on cardiometabolic, mental illness and related traits.

**Conclusions:**

Our results suggest that the associations for mental illness (and related traits) and those for cardiometabolic traits are distinct rather than shared and that these associations were consistent across ancestry groups.

**Clinical implications:**

Further investigation into the tissue-specific roles of *BMAL1* is required to fully understand the clinical impact of these findings.

**WHAT IS ALREADY KNOWN ON THIS TOPIC**

- The BMAL1 is a circadian rhythm regulator. Genetic variants in BAML1 are implicated in psychiatric disorders and cardiometabolic disease

**WHAT THIS STUDY ADDS**

- This is the first study to systematically assess shared vs distinct effect of this locus on mental and physical illness

**HOW THIS STUDY MIGHT AFFECT RESEARCH, PRACTICE OR POLICY**

- Using the UK Biobank study, we demonstrated that genetic effects on mental illness and cardiometabolic diseases are distinct (rather than shared) and are consistent across ancestries.
- Clinical implications of these findings are unclear, due to incomplete understanding of the tissue-specific roles of BMAL1.

## Background

 An association between cardiovascular disease and mental illness is well established. Patients with severe mental illness (SMI) are reported to have an increased risk of heart disease of between ~30% and 82%,^1^ with more SMI diagnoses being associated with increased risk (compared with no SMI, one condition increases risk by 50%, while five conditions increase risk by 300%).^2^

One hypothesis for the link between mental and cardiovascular illness is circadian rhythm dysregulation (such as shift work), which can negatively influence both mental and physical well-being. Evidence from human and mouse studies has shown that circadian rhythm dysregulation can lead to major depressive disorder (MDD) as well as increasing central fat deposition and risk of hypertension.^3^

Our study focused on a circadian rhythm regulation and control gene, *BMAL1*, which encodes the ARNTL protein (also called BMAL1, online supplemental figure 1), which forms a heterodimer with the CLOCK protein.^4^ This heterodimer promotes transcription of the PER1/2 and CRY1/2 proteins,^4^ which from CRY1/2:PER1/2 heterodimers, which inhibit ARNTL:CLOCK heterodimer transcription^4^ and regulate metabolic processes.^5^ The rate of transcription of *BMAL1* as well as production and breakdown of the ARNTL protein can set the period of the circadian rhythm.^4^

Murine studies have shown that *Bmal1* knockouts have reduced total sleep, non-REM sleep intensity and ability to recover from lost sleep,^6^ with stronger effects in mice with specific ablation of *Bmal1* in skeletal muscle, indicating a role of *Bmal1* expression outside the brain.^7^ *Bmal1* ablation in mice has also been associated with disrupted lipid and glucocorticoid metabolism.^8^ Mice with *Bmal1* overexpression exhibited circadian rhythm-dependent increases in lipid synthesis.^9^ Mouse studies have also indicated that *Bmal1* oscillation and periodicity is tissue dependent. Notably, disruptions to the internal oscillation period of the *Bmal1* gene in pancreatic islet tissue were associated with increased risk of type 2 diabetes (T2D) and obesity.^10^

In humans, genetic variation in *BMAL1* is robustly associated with neuropsychiatric disorders such as MDD, bipolar disorder (BD), seasonal affective disorder and schizophrenia^11 12^ as well as cardiometabolic phenotypes including hyperlipidaemia and coronary heart disease.^13 14^ However, it is unclear whether the same or separate *BMAL1* variants predispose mental and physical illnesses, as differences in populations used as well as methods and models applied mean that comparing effects between these studies is not straightforward. In addition, most studies have reported either mental illness or cardiometabolic associations, without considering both together.

## Objective

Our study aimed to describe the genetic architecture of the *BMAL1* locus, a regulator of circadian rhythm, in relation to psychiatric disorders, psychological/behavioural phenotypes and cardiometabolic diseases.

## Methods

### BMAL1 locus

The *BMAL1* locus was defined as the *BMAL1* coding gene±500 kb flanking the gene (chr11:13277790–13387266, UCSC Genome Browser).

### UK Biobank

UK Biobank (UKB) has been described previously in detail.^15 16^ In brief, between 2006 and 2010, ~500 000 participants were recruited through 22 centres across the UK. At baseline, participants completed extensive questionnaires on lifestyle, family medical history and personal medical history and medication use. A physical examination was conducted by trained staff, who recorded anthropometric measures and collected a blood sample for genetic and biochemical analyses. Participants self-reported ethnic background (data field #21000), which were categorised into broad ancestry groups (white British, white European, South Asian, African-Caribbean, Multiple/Other (henceforth referred to as Multiple)) as per Eastwood *et al*.^17^ Between 2016 and 2017, an online questionnaire was later sent to ~150 000 participants, with detailed questions on mental health. From 2015, a subset of participants was invited to undergo a range of imaging assessments (at the time of analyses, data were available for ~40 000 individuals).

### Ethical statement

All participants in UKB provided informed written consent and UKB was granted ethical approval by the NHS National Research Ethics Service on the 29 June 2021 (Ref 21/NW/0157). This study was conducted under project #71392, (P.I. Strawbridge).

### Phenotypes

Baseline variables included age at recruitment and sex, waist:hip ratio (WHR) (calculated from waist (#48) and hip circumferences (#49)), body mass index (BMI, #21001) (calculated by the central UKB team from height and weight measurements) and systolic and diastolic blood pressure (SBP (#4080) and DBP (#4079), respectively), which were calculated as the average of two readings. Self-reported history of ischaemic heart disease (ISH, including angina and heart attack, #6150), stroke (# 6150) and venous thromboembolism (VTE, #6152) and use of lipid-lowering (#6177 or 6153) or blood pressure (#6177 or 6153) medication. We also calculated blood pressure variables adjusted for use of blood pressure medication prior to measurement (SBPadj and DBPadj, respectively), as per Ehret *et al*^18^ T2D was defined as per Eastwood *et al* using self-report of diagnosis and medication use.^17^ Psychological and behavioural phenotypes were assessed via self-report for experience of anhedonia (#2060, binary variable defined as ever vs never), neuroticism score (#20127), mood instability (#1920), risk-taking (#2040), ever smoking (defined as ever vs never, #20160). Of note, neuroticism is recorded on a scale from 0 to 12 that reflects the number of ‘yes’ answers to 12 questions (the Eysenck Personality Questionnaire-Revised Short Form Neuroticism scale) and includes the mood instability variable. HbA1c levels (#30750) were also analysed.^19^

From the mental health questionnaire (Nmax=150 000) data used were self-reported any addiction (#20431) and lifetime experience of symptoms of BD, MDD and generalised anxiety disorder (GAD).^20^

From imaging data, ultrasound measurements of carotid intima-media thickness (IMT) were included; four measurements were recorded and three composite measures, namely average (IMT_mean_), maximum (IMT_max_) and average maximum (IMT_meanmax_) values computed.^21^ The three IMT measures were natural log transformed prior to analyses.

Where participants responded with ‘do not know’ or ‘prefer not to answer, data were omitted from analyses’.

### Genetic data

DNA extraction, genotyping and imputation were conducted by the central UKB team.^15^ Imputation was conducted using the Haplotype Reference Consortium (HRC) and the merged 1000 Genomes phase 3-UK10K reference panel (for variants not in the HRC) (Bycroft *et al*.^15^). Pre and postimputation quality control filters were applied by the central UKB team,^22^ whereby markers that had a missing rate of over 5% and a minor allele frequency (MAF) of less than 0.0001 were removed. Imputation info thresholds was dependent on MAF (Info >0.3 for MAF >3%, >0.6 for MAF 1%–3%, >0.8 for MAF 0.5%–1%, >0.9 for MAF 0.1%–0.5%).^22^ Standard quality control was also conducted by the central UKB team: participants were excluded if call rate <90%, sex chromosome aneuploidy, heterozygosity outliers and sex mismatch^15^; variants with call rates <95% were excluded. Genetic principal components (GPCs) were calculated centrally by the UKB team.^15^ One of each pair of related individuals (second cousins or closer) was excluded at random.

For each ancestry group, genetic variants within the *BMAL1* locus with MAF ≥1% were included. The number of variants available for each ancestry group were: 4232 for white British, 4213 for white European, 3980 for South Asian, 6356 for African-Caribbean and 4837 for multiple ancestries. For each ancestry group, Plink 1.09^23^ was used to determine how many variants were independent (using the indep-pairwise function with default settings) for each ancestry group: 936 for white British, 921 for white European, 759 for South Asian, 1864 for African-Caribbean and 1042 for multiple ancestries.

For lead variants of significant associations, linkage disequilibrium (LD) was visualised in Haploview, using r^2^ measures and default settings.^24^ A random subset of 50 000 white British participants were used, whereas for the other ancestry groups, the full dataset was used.

### Statistical analyses

Linear and logistic regression models were used in Plink 1.90^23^ to assess the association between genetic variants and phenotypes (BMI, WHR, HbA1c, T2D, SBPadj, DBPadj, IMT_mean_, IMT_max_, IMT_meanmax_, ISH, stroke, ever smoking, mood instability, anhedonia, neuroticism score, risk-taking behaviour, addiction, BD, MDD, GAD). An additive genetic model was assumed, and analyses were adjusted for age, sex, eight GPCs and genotyping chip (model 1). For analyses of VTE, BMI and ever smoking were also included. For analyses of IMT, ISH and stroke, ever-smoking, lipid-lowering and blood pressure medication were also included. LocusZoom (https://my.locuszoom.org/) was used for visualisation of association results, using the 1000 Genomes Nov 2014 EUR as the LD reference panel. Conditional analyses (inclusion of lead variant—defined as the variant with the lowest p value—as a covariate in analyses) were used for two purposes. First, the lead variant for Phenotype A was included as a covariate in analyses of phenotype A, to identify any secondary signals. Second, cross-trait analyses included the lead variant of phenotype A as a covariate in analyses of phenotype B, to investigate whether the signals were independent of each other. These results were interpreted alongside the LD visualisations.

Discovery analyses focused on white British participants, as these are the predominant group with greatest statistical power to detect associations. Bonferroni correction for multiple testing (number of independent variants) set the threshold for significance at p<5.35×10^−5^. Secondary analyses were conducted in additional ancestry data, for phenotypes with sufficient data (ie, baseline data only). Bonferroni-corrected significance thresholds were p<1.18×10^−5^ white European analyses, p<2.62×10^−5^ African-Caribbean analyses, p<6.58×10^−5^ South Asian analyses and p<4.09×10^−5^ multiple ancestry analyses.

Trans-ancestry meta-analyses were conducted in METAL,^25^ using inverse-variance weighted fixed effects. To assess trans-ancestry similarity, we focused on genetic variants present in ≥4 ancestry groups. Of 7336 genetic variants present in at least one ancestry group, 3793 variants were present in ≥4 groups, therefore significance was set at p<1.31×10^−5^ (ie, 0.05/3793) for all meta-analyses.

### In silico analyses

Lead variants were explored for genotype-specific gene expression levels, using the GTEx resource,^26^ and assessed for predicted functional effects using the Ensembl Variant Effect Predictor.^27^ The Online Mendelian Inheritance in Man (OMIM: https://omim.org/) website was used to explore the effects of rare variants in *BMAL1*, as rare and common variant phenotypes often converge.

## Findings

The characteristics of the UKB dataset (by self-reported broad ancestry group) are provided in table 1.

**Table 1:** Description of the UKB participants

	White British	White European	Multiple	South Asian	African-Caribbean
N (% men)	401 478 (46.0)	50 250 (43.8)	10 430 (42.8)	7656 (53.9)	7632 (43.0)
Age (years)	56.9 (8.0)	55.6 (8.1)	52.4 (8.1)	53.4 (8.5)	51.9 (8.1)
BMI (km/m^2^)	27.4 (4.8)	27.2 (4.9)	27.0 (4.9)	27.3 (4.5)	29.4 (5.4)
WHR	0.87 (0.09)	0.87 (0.09)	0.87 (0.08)	0.90 (0.09)	0.87 (0.08)
T2D	17 317 (4.3)	2105 (4.2)	865 (8.3)	1278 (16.7)	809 (10.6)
SBP	138 (19)	136 (19)	133 (19)	135 (19)	138 (19)
DBP	82 (10)	82 (10)	82 (11)	82 (10)	85 (11)
SBP*	141 (21)	138 (21)	136 (21)	139 (21)	143 (22)
DBP*	84 (11)	83 (11)	84 (12)	85 (11)	88 (12)
IMTmax	0.927 (0.210)				
IMTmean	0.690 (0.126)				
IMTmeanmax	0.802 (0.150)				
ISH	18 314 (6.1)	2122 (5.52)	414 (5.2)	569 (10.2)	263 (5.4)
Stroke	6124 (2.1)	703 (1.9)	94 (1.2)	127 (2.5)	118 (2.5)
VTE	10 379 (3.7)	1225 (3.5)	188 (2.8)	131 (2.4)	185 (3.6)
Lipid-lowering medication	69 919 (17.5)	8007 (16.1)	1807 (17.8)	2027 (27.4)	1208 (16.2)
Anti-hypertensive medication	83 666 (21.0)	9189 (18.5)	2048 (20.2)	2064 (27.9)	2336 (31.4)
Ever smoking	181 672 (45.4)	25 635 (51.2)	3987 (38.5)	1590 (21.0)	2279 (30.1)
Risk-taking	98 087 (25.3)	16 161 (33.7)	3698 (39.2)	2663 (39.5)	2983 (42.1)
Anhedonia	78 558 (20.2)	11 181 (23.2)	3333 (35.5)	2738 (42.8)	2382 (34.4)
Mood instability	177 338 (45.2)	21 967 (45.0)	4744 (48.6)	3737 (53.1)	3718 (52.2)
Neuroticism score	4.11 (3.25)	4.16 (3.30)	4.12 (3.39(	4.39 (3.48)	3.68 (3.20)
Completed the MHQ	130 050 (32.4)	16 664 (33.2)	2273 (21.8)	1026 (13.4)	1103 (14.5)
Addiction	7447 (5.8)	1273 (7.7)	165 (7.4)	46 (4.5)	64 (5.83)
LifeGAD	9068 (10.1)	1272 (11.5)	166 (10.9)	73 (10.4)	54 (6.7)
LifeBD	1866 (1.4)	321 (1.9)	51 (2.3)	23 (2.3)	24 (2.2)
LifeMDD	30 789 (37.0)	4156 (39.8)	538 (39.0)	191 (29.8)	203 (28.8)

Where: unless otherwise stated, data are presented as N % or mean (SD). MHQ, mental health questionnaire.

BD, bipolar disorder; BMI, body mass index; DBP, diastolic blood pressure; GAD, generalised anxiety disorder; IMT, intima-media thickness; ISH, ischaemic heart disease; MDD, major depressive disorder; SBP, systolic blood pressure; T2D, type 2 diabetes; VTE, venous thromboembolism; WHR, waist:hip ratio.

### BMAL1 variants in white British individuals

For mental illness phenotypes, *BMAL1* variants were significantly associated (table 2, online supplemental figure 2) with anhedonia (rs34862781-G, OR 1.03 (95% CI 1.01 to 1.04), p=2.66×10^−5^, online supplemental table 1, online supplemental figure 2A), neuroticism (rs745752200-ATG, Beta 0.055 (SE 0.008, online supplemental table 2, online supplemental figure 2B), p=1.11×10^−11^), ever smoking (rs5789783-T, 1.02 (1.01–1.03), p=4.21×10^−5^, online supplemental table 3, online supplemental figure 2C) and risk-taking behaviour (rs12419833-T, 1.03 (1.01–1.04), p=4.96×10^−6^, online supplemental table 4, online supplemental figure 2D). No additional signals were found and no significant associations were identified for addiction, BD, GAD, MDD or mood instability.

**Table 2:** Lead variants in single ancestry analyses

Analysis	Signal	Chr	SNP	BP	A1	A2	MAF	N	OR	L95	U95	P	Beta	SE	P	N sig
White British	Anhedonia	11	rs34862781	13305263	G	A	0.42	387 549	1.03	1.01	1.04	2.66E-05				1
	Neuroticism	11	rs745752200	13279018	ATG	A	0.42	325 313					0.055	0.008	1.14E-11	214
	Ever smoking	11	rs5789783	13347748	T	TA	0.40	343 531	1.02	1.01	1.03	4.21E-05				1
	Risk-taking	11	rs12419833	13235881	T	A	0.46	385 880	1.03	1.01	1.04	4.96E-06				15
	DBPadj	11	rs10832013	13295353	G	T	0.30	398 187					−0.216	0.027	7.68E-16	228
	SBPadj	11	rs900144	13294268	C	T	0.43	398 261					−0.307	0.043	8.28E-13	205
	BMI1	11	rs11826177	13349477	T	C	0.36	380 161					−0.086	0.011	2.01E-14	214
	BMI2	11	rs12802633	13055517	A	C	0.08	392 116					0.087	0.020	1.28E-05	
	WHR1	11	rs570129974	13299258	A	AG	0.34	379 162					−0.001	0.000	2.43E-10	340
	WHR1 proxy	11	rs61882122	13299895	A	G	0.30	398 813					−0.001	0.000	1.01E-08	
	WHR2	11	11:13 356 159	13356159	T	TAGA	0.02	396 613					0.003	0.001	1.40E-07	
	WHR2 proxy	11	rs12790791	13830128	A	G	0.08	401 225					0.001	0.000	6.30E-06	
	WHR3	11	rs10832027	13357183	G	A	0.31	400 554					−0.001	0.000	3.19E-10	
	HbA1c	11	rs16913144	13798118	A	C	0.16	364 176					0.057	0.014	4.82E-05	3
White European	DBPadj	11	rs7112233	13255971	T	C	0.22	55 837					−0.422	0.080	1.51E-07	103
African-Caribbean	Mood instability	11	rs141886574	13106882	T	C	0.02	6971	1.74	1.35	2.24	2.06E-05				1
	T2D	11	rs115057388	13584056	T	C	0.02	7468	2.14	1.53	2.99	1.02E-05				3
Multiple ancestries	HbA1c1	11	rs16911697	12893577	G	A	0.02	8725					1.660	0.358	3.48E-06	3
	HbA1c2	11	rs114831190	13177079	C	T	0.02	8708					1.519	0.345	1.08E-05	
	WHR	11	rs11022734	13281580	A	G	0.39	9740					−0.004	0.001	3.75E-05	3

Significance was set a p<5.35x10-5 for white British, p<1.18x10-5 for white European analyses, p<2.62x10-5 for African-Caribbean analyses, 6.58x10-5 for South Asian analyses and p<4.09x10-5 for multiple ancestry analyses. SNP, single nucleotide polymorphism; BP, base position; HbA1c, glycated haemaglobin; WHR1 and WHR2, WHR signal 1 and WHR signal 2.

A1, effect allele/minor allele; A2, non-effect allele; BMI, body mass index; MAF, minor/effect allele frequency; N sig, number of significant variants; T2D, type 2 diabetes; WHR, waist:hip ratio.

For cardiometabolic traits, *BMAL1* variants were significantly associated (table 2, online supplemental figure 2) with DBPadj (rs10832013-G, −0.216 (0.027), p=7.68×10^–16^, online supplemental table 5, online supplemental figure 2E), SBPadj (rs90144-C, −0.307 (0.043), p=8.28×10^–13^, online supplemental table 6, online supplemental figure 2F)), and BMI (rs11826177-T, −0.086 (0.011), p=2.01×10^-–14^, online supplemental table 7, online supplemental figure 2G). Conditional analyses identified a further signal for BMI (BMI2, rs12802633-A, 0.087 (0.020), p=1.28×10^–5^). The lead variant for WHR (rs570129974-A, −0.001 (0.000), p=2.43×10^–10^, online supplemental table 8) was not present in the LocusZoom reference panel, therefore the neighbouring significant variant rs10832027 was plotted instead (online supplemental figure 2H). Conditional analyses identified two additional signals for WHR (WHR2, 11:13356159, 0.003 (0.001), p=1.40×10^–7^). This variant was not possible to plot, therefore rs12790791 was plotted (online supplemental figure 2I) and rs1083207-G, −0.001 (0.000), p=3.19×10^–10^, (online supplemental figure 2J). HbA1c levels also showed significant associations (rs16913144-A, 0.057 (0.014), p=4.82×10^−5^, online supplemental table 9, online supplemental figure 2K).

### Cross-trait analyses

If two genetic variants are in high LD (highly correlated) and therefore represent the same genetic signal, then adjusting one association for the other lead variant would be expected to render the association null (both in terms of the P and effect size).

LD between lead variants in white British ancestry individuals (online supplemental figure 3) demonstrated that the risk-taking lead variant is not strongly correlated with any other lead variant (highest LD r^2^=0.44), however, adjustment for the lead variants for anhedonia, BMI1, smoking, neuroticism, SBP and WHR1 rendered the associations non-significant but had little impact on the effect size (online supplemental table 4).

The signals for anhedonia and neuroticism were strongly correlated (r^2^=0.82), and cross-trait conditional analyses were no longer significant (online supplemental tables 1 and 2). There was only moderate correlation of the anhedonia and neuroticism signals with the signal for ever smoking (r^2^=0.65 and 0.56, respectively), however, adjustment for the smoking lead rendered the associations null, but with little impact on the effect size (online supplemental tables 1 and 2). Similarly, the smoking association became null after adjustment for the anhedonia or neuroticism leads (online supplemental table 3).

The proxy used in the regional plots for WHR1 (online supplemental figure 3) was appropriate (r^2^=0.81). In contrast, the proxy used for WHR2 was not (r^2^≤0.1). BMI1 demonstrated moderate correlation with WHR1 (r^2^=0.50) and low correlation with WHR3 (r^2^=0.33), but no correlation with WHR2. BMI2 demonstrated no correlation with the WHR signals. After cross-trait conditional analyses with any other lead variants, associations with BMI were still significant (online supplemental table 7). Associations with WHR1 (but not WHR2) were null after adjustment for the anhedonia, BMI1, DBP, SBP, smoking and neuroticism leads, however, effect sizes were unchanged (online supplemental table 8).

The DBPadj and SBPadj leads were moderately correlated (r^2^=0.57), and these were moderately correlated with DBPadj and SBPadj (r^2^=0.39 and 0.63, respectively) or WHR1 (r^2^=0.79 and 0.73), but not with the WHR2 and WHR3 signals. Cross-trait conditional analyses with WHR1 and SBP removed the association with DBP, but with little impact on the effect size (online supplemental table 5). Cross-trait conditional analyses with BMI1, WHR1 and DBP gave non-significant associations with SBP, again with little impact on effect sizes (online supplemental table 6). The signal for HbA1c appeared to be independent of all other signals, however, cross-trait analyses (namely BMI1, WHR1, DBP1, smoking, neuroticism and risks) with removed the significance of the association but had little impact on effect size (online supplemental table 9).

The lead variants for neuroticism or anhedonia and SBPadj appear to be strongly correlated (r^2^=0.87 and 0.94, respectively) (online supplemental figure 3); again, the cross-trait associations were no longer significant in the conditional analyses but with little impact on effect size. Similarly, the lead variants for BMI1 and ever smoking suggested a shared signal (r^2^=0.90), but cross-trait conditional analyses were not consistent with this.

### BMAL1 variants in white European individuals

For mental illness phenotypes, no significant associations were observed.

For cardiometabolic traits, a single significant association was identified for DBPadj (rs7112233-T, −0.422 (0.080), p=1.51×10^−7^, table 2, online supplemental figure 4A, online supplemental table 10).

### BMAL1 variants in south Asian individuals

No significant associations were identified for mental illness or cardiometabolic traits (lowest p=1.29×10^−4^).

### BMAL1 variants in African-Caribbean individuals

For mental illness phenotypes, a single significant association was observed for mood instability (rs141886574-T, 1.74 (1.36–2.24), p=2.06×10^−5^, table 2, online supplemental figure 4, online supplemental table 11).

For cardiometabolic traits, a significant association was identified for T2D (rs115057388-T, 2.14 (1.53–3.00), p=1.02×10^−5^, table 2, online supplemental figure 4C, online supplemental table 12).

Cross-trait analyses did not support the signals for T2D and mood instability being shared (no impact on p or effect sizes)

### BMAL1 variants in multiple ancestry individuals

No significant associations were identified for mental illness phenotypes.

For cardiometabolic traits, a significant association was observed with HbA1c (rs16911697-G, 1.66 (0.358), p=3.48×10^−6^, table 2, online supplemental figure 4D, online supplemental table 13). Conditional analyses identified a further signal (rs114831190-C, 1.519 (0.345), p=1.08×10^−5^, table 2, online supplemental figure 4E, online supplemental table 13). A significant association was also identified for WHR (rs11022734-A, −0.004 (0.001), p=3.75×10^−5^, table 2, online supplemental figure 4F, online supplemental table 14).

Genetic associations with WHR were unchanged when HbA1c lead variants were included as covariates. In contrast, the effect of one genetic variant (rs114831190, HbA1c2) on HbA1c is notably reduced when adjusting for WHR1 (online supplemental table 14).

### Trans-ancestry meta-analyses

Heterogeneity (represented by Isq) reflects the proportion of the observed signal that might be due to variability between the ancestry groups. No heterogeneity suggests common effects across ancestries. Table 3 provides the lead variants for significant associations (most significant, as well as most significant with Isq=0).

**Table 3:** Lead variants in the trans-ancestry meta-analyses

Phenotype	Chr	Pos	SNP	A1	A2	N	Freq A1	Freq SE	Min freq	Max freq	Beta	SE	P value	Direction	HetISq	HetPVal	N sig	N Isq=0	% Isq=0
Neuroticism	11	13279979	rs55689167	A	G	379 542	0.57	0.04	0.06	0.58	−0.054	0.008	4.86E-13	--++-	0	0.422	113	49	43
	11	13279018	rs745752200	A	ATG	387 169	0.57	0.04	0.07	0.58	−0.054	0.007	6.16E-13	--++-	29.2	0.227			
Risk-taking	11	13238235	rs12788850	A	G	454 836	0.46	0.03	0.06	0.51	0.022	0.005	7.90E-06	++-++	0	0.651	7	7	100
T2D	11	13228736	rs10734211	A	C	477 451	0.78	0.07	0.56	0.81	0.053	0.012	6.22E-06	+++++	51.8	0.081	1	0	0
DBP	11	13295353	rs10832013	T	G	478 962	0.69	0.05	0.23	0.7	0.222	0.024	9.91E-20	++-++	15.4	0.316	243	38	16
	11	13291823	rs148728593	A	ATATATT	478 195	0.3	0.03	0.29	0.47	−0.222	0.025	1.71E-19	--+--	0	0.494			
SBP	11	13288885	rs61882109	A	C	479 137	0.44	0.04	0.43	0.92	−0.286	0.039	3.68E-13	--++-	47.6	0.106	223	46	21
	11	13347748	rs5789783	T	TA	409 004	0.41	0.05	0.4	0.9	−0.306	0.043	1.23E-12	--++-	0	0.629			
BMI	11	13349477	rs11826177	T	C	452 832	0.37	0.03	0.36	0.49	−0.076	0.010	1.71E-13	-----	21.8	0.276	211	22	10
	11	13248730	rs35424804	T	C	482 296	0.49	0.03	0.06	0.5	0.055	0.010	1.37E-08	++--+	0	0.537			
WHR	11	13274553	rs747601	A	T	479 339	0.28	0.03	0.27	0.48	−0.001	0.000	1.75E-10	--+--	73.3	0.005	315	5	2
	11	13355770	rs6486121	T	C	480 771	0.63	0.05	0.42	0.64	0.001	0.000	6.57E-10	++-++	0	0.478			

A1, allele; A2, allele 2; BMI, body mass index; DBP, diastolic blood pressure; Direction, direction of effect in White British, White European, South Asian, African Caribbean, multiple ancestries; Freq1, allele 1 frequency; Het Isq, heterogeneity I2; HetPVal, heterogeneity p value; %Isq=0, % of significant variants with Isq=0; NIsq=0, number of significant variants with Is=0; Nsig, number of significant variants; SBP, systolic blood pressure; T2D, type 2 diabetes; WHR, waist:hip ratio.

**Figure 1:**
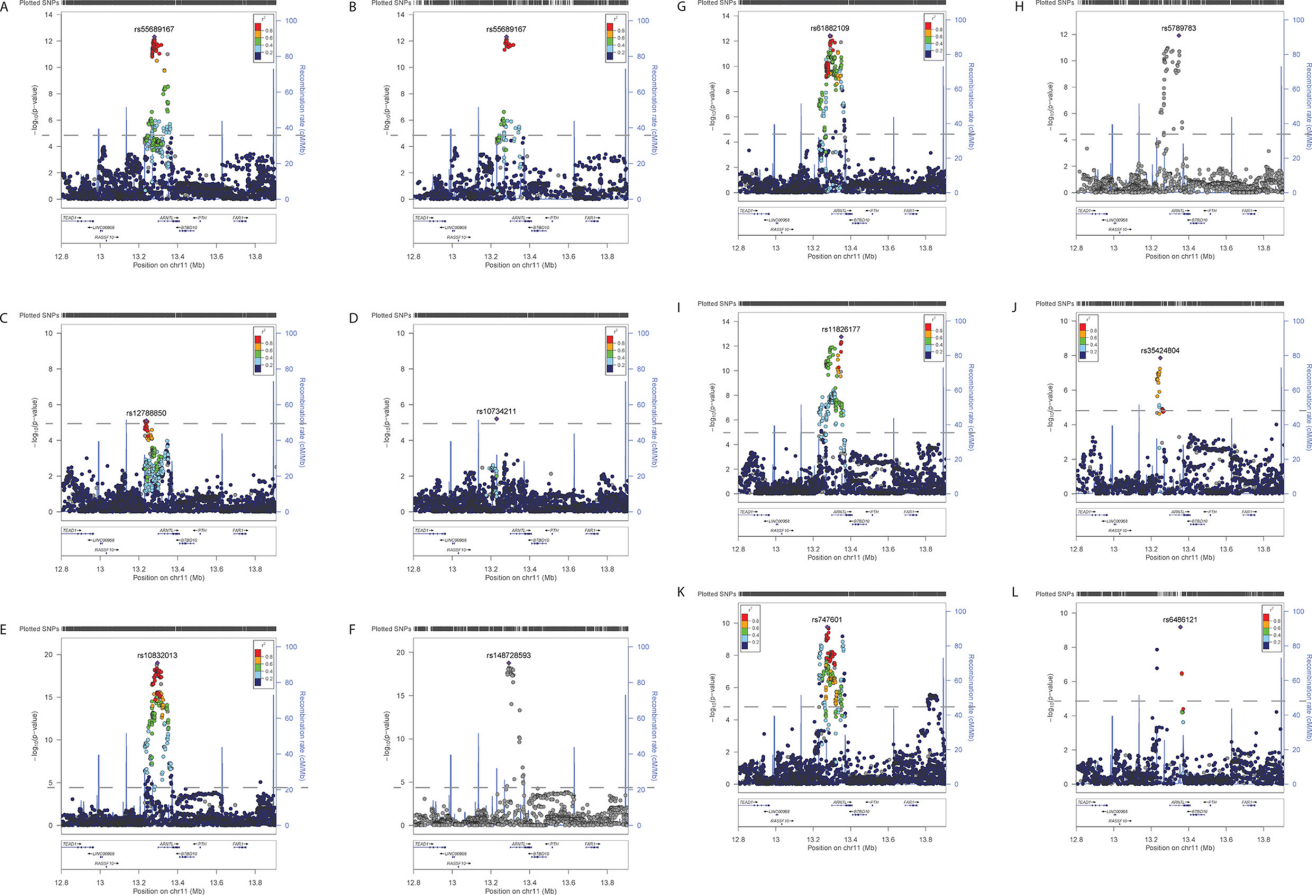
Regional association plots of meta-analyses results of (A) neuroticism score, (**B**) neuroticism score (Isq=0), (**C**) risk-taking, (**D**) T2D, (**E**) DBPadj, (**F**) DBPadj (Isq=0), (**G**) SBPadj, (**H**) SBPadj (Isq=0), (**I**) BMI, (**J**) BMI (Isq=0), (**K**) WHR, (**L**) WHR (Isq=0). The lead variant is represented by a purple diamond. The other variants are colours according to LD r^2^ values. The horizontal dashed line represents the Bonferonni-corrected threshold for significance. BMI, body mass index; DBP, diastolic blood pressure; SBP, systolic blood pressure; T2D, type 2 diabetes; WHR, waist:hip ratio.

For mental illness phenotypes, significant associations were observed for neuroticism score, of which 43% had no heterogeneity (table 3, figure 1A,B, online supplemental table 15). Seven variants were significantly associated with risk-taking, all with no heterogeneity (Isq=0, table 3, figure 1C, online supplemental table 16).

For cardiometabolic traits, only one variant was significantly associated with T2D and that demonstrated moderate heterogeneity (Isq=51.8, table 3, figure 1D, online supplemental table 17). Significant associations were observed for DBPadj, of which 16% showed no heterogeneity (table 3, figure 1E,F, online supplemental table 18), and SBPadj, of which 21% had no heterogeneity (table 3, figure 1G,H, online supplemental table 19). Significant associations were identified for BMI, but only 10% of these showed no heterogeneity (table 3, figure 1I,J, online supplemental table 20), and for WHR, but only 2% of these had no heterogeneity (5 of 315, table 3, figure 1K,L, online supplemental table 21).

For all traits except T2D, there were significant associations where heterogeneity did not contribute (where I^2^=0), suggesting consistent effects across ancestry groups.

No genetic variants met the threshold for significance for anhedonia (online supplemental table 22), mood instability (online supplemental table 23), ever smoking (online supplemental table 24) or HbA1c (online supplemental table 25).

### Trans-ancestry LD plots

The LD patterns between all lead variants (single ancestry analyses or meta-analyses) are presented for white British (online supplemental figure 3), white European (online supplemental figure 5), South Asian (online supplemental figure 6), African-Caribbean (online supplemental figure 7) and multiple ancestry individuals (online supplemental figure 8).

African ancestry populations are particularly useful for interpreting shared versus distinct signals, due to the typically shorter LD blocks, therefore we focused on the African-Caribbean ancestry data (online supplemental figure 7). The African-Caribbean ancestry LD patterns demonstrate that the risk-taking signal identified in the meta-analysis is the same as that identified in the white British ancestry group (LD r^2^=0.99), and that this signal is shared with the meta-analysis BMI lead with low heterogeneity (r^2^=0.81). The neuroticism lead variants (in white British ancestry or meta-analyses) represent the same signal (r^2^=0.82). The WHR signals identified in the Multiple ancestry group and the meta-analysis represent the same signal (r^2^=0.81). The meta-analysis SBPadj lead variant and that from the white British analysis represent the same signal (r^2^=0.95), and there is moderate LD between the SBPadj and neuroticism signals (r^2^=0.56–0.73).

### Follow-up analyses

GTEx data demonstrate that *BMAL1* (*ARNTL* in GTEx) was expressed at low levels in all tissues (online supplemental figure 10). Of the lead variants, 13 had genotype-specific effects on *BMAL1* gene expression (online supplemental table 26) and most were in whole blood (online supplemental table 27).

Most of the lead variants were predicted to have low functional impact. Three variants were predicted to impact transcription factor binding (online supplemental table 27), but it is unclear what impact this would have (positive or negative) on gene expression.

OMIM reports no evidence for rare variants in the *BMAL1* locus, suggesting an important role for this protein (as deleterious variants are selected against).

## Discussion

This study systematically investigated common genetic variation in the *BMAL1* locus a range of phenotypes, to assess whether this circadian rhythm gene might represent a biological mechanism linking mental illness to cardiometabolic diseases, using data from the UKB population study.

We identified associations between genetic variation in *BMAL1* and cardiometabolic (HbA1c, DBP, SBP, ever smoking, BMI and WHR) and mental illness phenotypes (anhedonia, MDD, risk-taking behaviour and neuroticism). Meta-analyses indicated that for most phenotypes, there were consistent effects across ancestries. Analyses investigating potential shared signals provided mainly inconclusive evidence. However, further investigation into the associations between SBP and psychological traits (anhedonia and neuroticism) would be of interest.

These associations are consistent with previous studies: rs10832013-G (DBP) was previously associated with reduction in FEV1/FVC,^13^ rs5789783-T (ever smoking) with increased risk of metabolic syndrome (defined as a combination of diabetes, obesity and cardiovascular disease^14^ and rs900144-C (SBP) with increased BMI).^13^ Furthermore, in line with the well-documented inflammatory effects of increased adipose storage, rs10832027-G (WHR) was associated with decreased C reactive protein, which is a measure of inflammatory response.^28^

Animal studies suggest that altered *Bmal1* expression levels affect circadian rhythm and metabolic phenotypes, likely in a tissue-specific manner. Consistent with this, we see positive and negative associations between increased *BMAL1* gene expression in blood and metabolic or psychiatric phenotypes. Further research is required to understand the tissue-specific mechanisms behind *BMAL1* associations with human disease.

The cross-trait analyses provided inconclusive evidence, with changes in effect sizes and significance not being consistent with LD patterns. However, the possibility of shared genetic signals for SBP, anhedonia and neuroticism is worth further consideration (alternative methods might be better are resolving this issue). Additional studies in larger samples of non-European ancestry would help draw more definitive conclusions about the overlap between signals.

Our analyses have limitations: UKB is a large and consistently phenotyped cohort, but does not reflect the general population of the UK, with participants being predominantly white British and are healthier and wealthier than the UK average.^29^ We acknowledge that self-reported and genetically defined ancestry do not fully overlap, and the ongoing discussion about how best to appropriately account for population structure.^30^ It is clear that continental-based ‘ancestry’ grouping is suboptimal, however this is a pragmatic approach based on the questions asked of participants by UKB.

In summary, this study demonstrated associations between the *BMAL1* locus and psychological, behavioural, cardiovascular and metabolic phenotypes, with consistent effects across ancestries. Our findings provided little evidence to support the hypothesis that *BAML1* might represent a mechanism linking mental and physical health.

## Clinical implications

Further investigation into the tissue-specific roles of *BMAL1* is required to fully understand the clinical impact of these findings.

## Supplementary material

10.1136/bmjment-2024-301267 online supplemental file 1

10.1136/bmjment-2024-301267 online supplemental table 1

## Data Availability

Data may be obtained from a third party and are not publicly available.

## References

[1] De Hert M, Detraux J, Vancampfort D (2018). The intriguing relationship between coronary heart disease and mental disorders. Dialogues Clin Neurosci.

[2] Scott KM, de Jonge P, Alonso J (2013). Associations between DSM-IV mental disorders and subsequent heart disease onset: beyond depression. Int J Cardiol.

[3] Walker WH, Walton JC, DeVries AC (2020). Circadian rhythm disruption and mental health. Transl Psychiatry.

[4] Buhr ED, Takahashi JS (2013). Molecular components of the Mammalian circadian clock. Handb Exp Pharmacol.

[5] Ramsey KM, Yoshino J, Brace CS (2009). Circadian clock feedback cycle through NAMPT-mediated NAD+ biosynthesis. Science.

[6] Laposky A, Easton A, Dugovic C (2005). Deletion of the mammalian circadian clock gene BMAL1/Mop3 alters baseline sleep architecture and the response to sleep deprivation. Sleep.

[7] Ehlen JC, Brager AJ, Baggs J (2017). Bmal1 function in skeletal muscle regulates sleep. Elife.

[8] Leliavski A, Shostak A, Husse J (2014). Impaired glucocorticoid production and response to stress in Arntl-deficient male mice. Endocrinology.

[9] Shimba S, Ishii N, Ohta Y (2005). Brain and muscle Arnt-like protein-1 (BMAL1), a component of the molecular clock, regulates adipogenesis. Proc Natl Acad Sci U S A.

[10] Marcheva B, Ramsey KM, Buhr ED (2010). Disruption of the clock components CLOCK and BMAL1 leads to hypoinsulinaemia and diabetes. Nature New Biol.

[11] Lamont EW, Legault-Coutu D, Cermakian N (2007). The role of circadian clock genes in mental disorders. Dialogues Clin Neurosci.

[12] Liu JJ, Sudic Hukic D, Forsell Y (2015). Depression-associated ARNTL and PER2 genetic variants in psychotic disorders. Chronobiol Int.

[13] Kichaev G, Bhatia G, Loh P-R (2019). Leveraging Polygenic Functional Enrichment to Improve GWAS Power. Am J Hum Genet.

[14] Lind L (2019). Genome-Wide Association Study of the Metabolic Syndrome in UK Biobank. Metab Syndr Relat Disord.

[15] Bycroft C, Freeman C, Petkova D (2018). The UK Biobank resource with deep phenotyping and genomic data. Nature New Biol.

[16] Sudlow C, Gallacher J, Allen N (2015). UK biobank: an open access resource for identifying the causes of a wide range of complex diseases of middle and old age. PLoS Med.

[17] Eastwood SV, Mathur R, Atkinson M (2016). Algorithms for the Capture and Adjudication of Prevalent and Incident Diabetes in UK Biobank. PLoS One.

[18] Ehret GB, Ferreira T, Chasman DI (2016). The genetics of blood pressure regulation and its target organs from association studies in 342,415 individuals. Nat Genet.

[19] Ranglani S, Ward J, Sattar N (2023). Testing for associations between HbA1c levels, polygenic risk and brain health in UK Biobank (N = 39 283). Diabetes Obes Metab.

[20] Davis KAS, Coleman JRI, Adams M (2020). Mental health in UK Biobank - development, implementation and results from an online questionnaire completed by 157 366 participants: a reanalysis. BJPsych Open.

[21] Strawbridge RJ, Ward J, Bailey MES (2020). Carotid Intima-Media Thickness: Novel Loci, Sex-Specific Effects, and Genetic Correlations With Obesity and Glucometabolic Traits in UK Biobank. Arterioscler Thromb Vasc Biol.

[22] Mitchell RE, Hemani G, Dudding T (2019). UK biobank genetic data: MRC-IEU quality control, version 2. https://data.bris.ac.uk/datasets/1ovaau5sxunp2cv8rcy88688v/UK%20Biobank%20Genetic%20Data_MRC%20IEU%20Quality%20Control%20version%202.pdf.

[23] Chang CC, Chow CC, Tellier LC (2015). Second-generation PLINK: rising to the challenge of larger and richer datasets. Gigascience.

[24] Barrett JC, Fry B, Maller J (2005). Haploview: analysis and visualization of LD and haplotype maps. Bioinformatics.

[25] Willer CJ, Li Y, Abecasis GR (2010). METAL: fast and efficient meta-analysis of genomewide association scans. Bioinformatics.

[26] Lonsdale J, Thomas J, Salvatore M (2013). The Genotype-Tissue Expression (GTEx) project. Nat Genet.

[27] McLaren W, Gil L, Hunt SE (2016). The Ensembl Variant Effect Predictor. Genome Biol.

[28] Ligthart S, Vaez A, Võsa U (2018). Genome Analyses of >200,000 Individuals Identify 58 Loci for Chronic Inflammation and Highlight Pathways that Link Inflammation and Complex Disorders. Am J Hum Genet.

[29] Fry A, Littlejohns TJ, Sudlow C (2017). Comparison of Sociodemographic and Health-Related Characteristics of UK Biobank Participants With Those of the General Population. Am J Epidemiol.

[30] National Academies of Sciences E, and Medicine; Division of Behavioral and Social Sciences and Education; Health and Medicine Division; Committee on Population; Board on Health Sciences Policy; Committee on the Use of Race, Ethnicity, and Ancestry as Population Descriptors in Genomics Research (2023). Using Population Descriptors in Genetics and Genomics Research: A New Framework for an Evolving Field.

