# Supplementary material for: Genetic variation in circadian regulator gene BMAL1 in psychiatric, psychological and cardiometabolic traits: a trans-ancestry UK Biobank study
Source: BMJ Ment Health. 2024 Dec 12;27(1):e301267. doi: 10.1136/bmjment-2024-301267 (PMC11647332; doi:10.1136/bmjment-2024-301267)
Supplement: online supplemental file 1 [file bmjment-27-1-s001.pdf]

Genetic variation in *BMAL1* in psychiatric disorders and cardiometabolic diseases: a trans-ancestry UK Biobank study

Supplementary Data

Supplementary Figure 1: A diagram describing the basic function of the *BMAL1* gene product, ARNTL, through its interaction with other genes and proteins. Green arrows indicate up-regulation and red arrows indicate down-regulation.

Supplementary Figure 2: Regional plots for white British analyses of A) anhedonia, B) neuroticism score, C) ever smoking, D) risk-taking behaviour, E) DBPadj, F) SBPadj, G) HbA1c levels, H) BMI1, I) BMI2 (where the BMI1 was included as a covariate in the analyses), J) WHR1, K) WHR2 (where the WHR1 was included as a covariate in the analyses) and WHR3 (where WHR1 and WHR2 were included as covariates). Of note, rs61882122 is a proxy for WHR1 and rs12790291 is a proxy for WHR2.

Supplementary Figure 3: LD patterns of all lead variants (with MAF>1%) in the white British ancestry group. LD colours and figures are r2. Black text, white British lead variant. Blue text, white European lead variant. Grey text, African-Caribbean lead variant. Yellow, multiple ancestries lead variant. Magenta, meta-analysis lead variant.

Supplementary Figure 4: Regional plots of A) white European DBPadj, B) African-Caribbean mood instability C) African-Caribbean T2D, D) multiple ancestry HbA1C analyses, E) multiple ancestry HbA1C analyses second independent signal and F) multiple ancestries WHR.

Supplementary Figure 5: LD patterns of all lead variants (with MAF>1%) in the white European ancestry group. LD colours and figures are r2. Black text, white British lead variant. Blue text, white European lead variant. Grey text, African-Caribbean lead variant. Yellow, multiple ancestries lead variant. Magenta, meta-analysis lead variant.

Supplementary Figure 6: LD patterns of all lead variants (with MAF>1%) in the South Asian ancestry group. LD colours and figures are r2. Black text, white British lead variant. Blue text, white European lead variant. Grey text, African-Caribbean lead variant. Yellow, multiple ancestries lead variant. Magenta, meta-analysis lead variant.

Supplementary Figure 7: LD patterns of all lead variants (with MAF>1%) in the African-Caribbean ancestry group. LD colours and figures are r2. Black text, white British lead variant. Blue text, white European lead variant. Grey text, African-Caribbean lead variant. Yellow, multiple ancestries lead variant. Magenta, meta-analysis lead variant.

Supplementary Figure 8: LD patterns of all lead variants (with MAF>1%) in the Multiple ancestries group. LD colours and figures are r2. Black text, white British lead variant. Blue text, white European lead variant. Grey text, African-Caribbean lead variant. Yellow, multiple ancestries lead variant. Magenta, meta-analysis lead variant.

Supplementary Figure 9: GTEx BMAL1 gene expression data by tissue.

Supplementary Table 1: Analyses and conditional analyses of anhedonia in white British group

Supplementary Table 2: Analyses and conditional analyses of neuroticism score in white British group

Supplementary Table 3: Analyses and conditional analyses of ever smoking in white British group

Supplementary Table 4: Analyses and conditional analyses of risk taking in white British group

Supplementary Table 5: Analyses and conditional analyses of DBP in white British group

Supplementary Table 6: Analyses and conditional analyses of SBP in white British group

Supplementary Table 7: Analyses and conditional analyses of BMI in white British group

Supplementary Table 8: Analyses and conditional analyses of WHR in white British group

Supplementary Table 9: Analyses and conditional analyses of HbA1C in white British group

Supplementary Table 10: Analyses and conditional analyses of DBP in white European group

Supplementary Table 11: Analyses and conditional analyses of mood instability in African-Caribbean group

Supplementary Table 12: Analyses and conditional analyses of T2D in African-Caribbean group

Supplementary Table 13: Analyses and conditional analyses of HbA1c in the multiple ancestry group

Supplementary Table 14: Analyses and conditional analyses of WHR in the multiple ancestry group

Supplementary Table 15: meta-analysis of neuroticism score

Supplementary Table 16: meta-analysis of risk-taking

Supplementary Table 17: meta-analysis of DBP

Supplementary Table 18: meta-analysis of SBP

Supplementary Table 19: meta-analysis of T2D

Supplementary Table 20: meta-analysis of BMI

Supplementary Table 21: meta-analysis of WHR

Supplementary Table 22: meta-analysis of anhedonia

Supplementary Table 23: meta-analysis of mood instability

Supplementary Table 24: Meta-analysis of ever smoking

Supplementary Table 25: Meta-analysis of HbA1c

Supplementary Table 26: Availability of lead variants in GTEx and eQTL status.

Supplementary Table 27: EQTLs for lead variants in GTEx. Of note, positions here are build 38 (whereas results data are b37).

Supplementary Table 28: Variants with predicted impact on transcription factor binding.

Figure 1

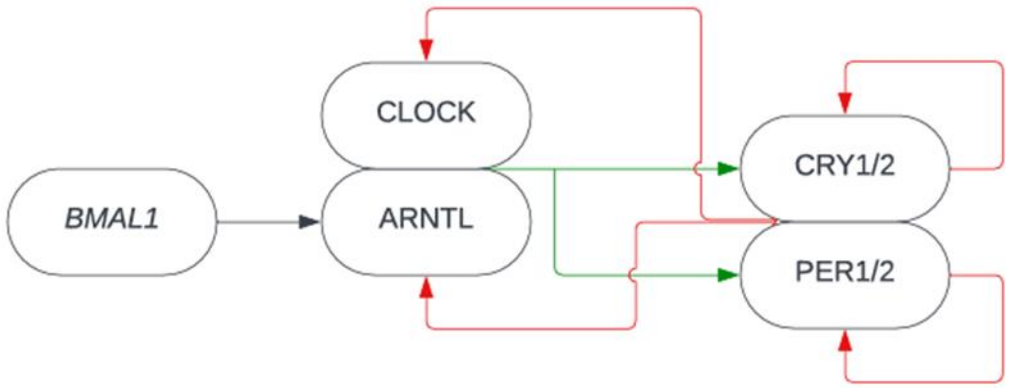

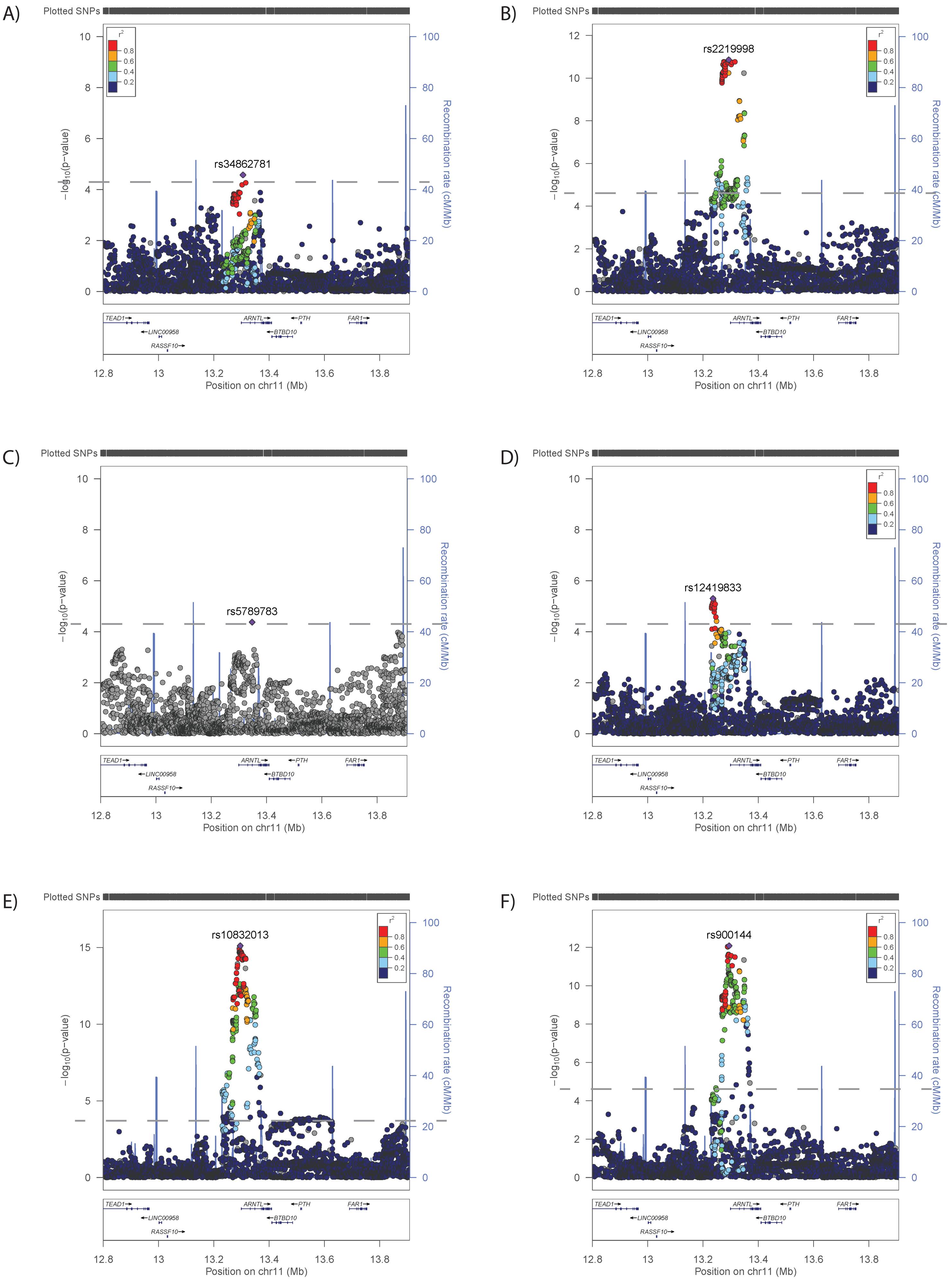
A)
Plotted SNPs
rs34862781
B)
Plotted SNPs
rs2219998
C)
Plotted SNPs
rs5789783
D)
Plotted SNPs
rs12419833
E)
Plotted SNPs
rs10832013
F)
Plotted SNPs
rs900144
r²
0.8
0.6
0.4
0.2
−log10(p−value)
Recombination rate (cM/Mb)
TEAD1
LINC00958
RASSF10
ARNTL
BTBD10
PTH
FAR1
12.8
13
13.2
13.4
13.6
13.8
Position on chr11 (Mb)

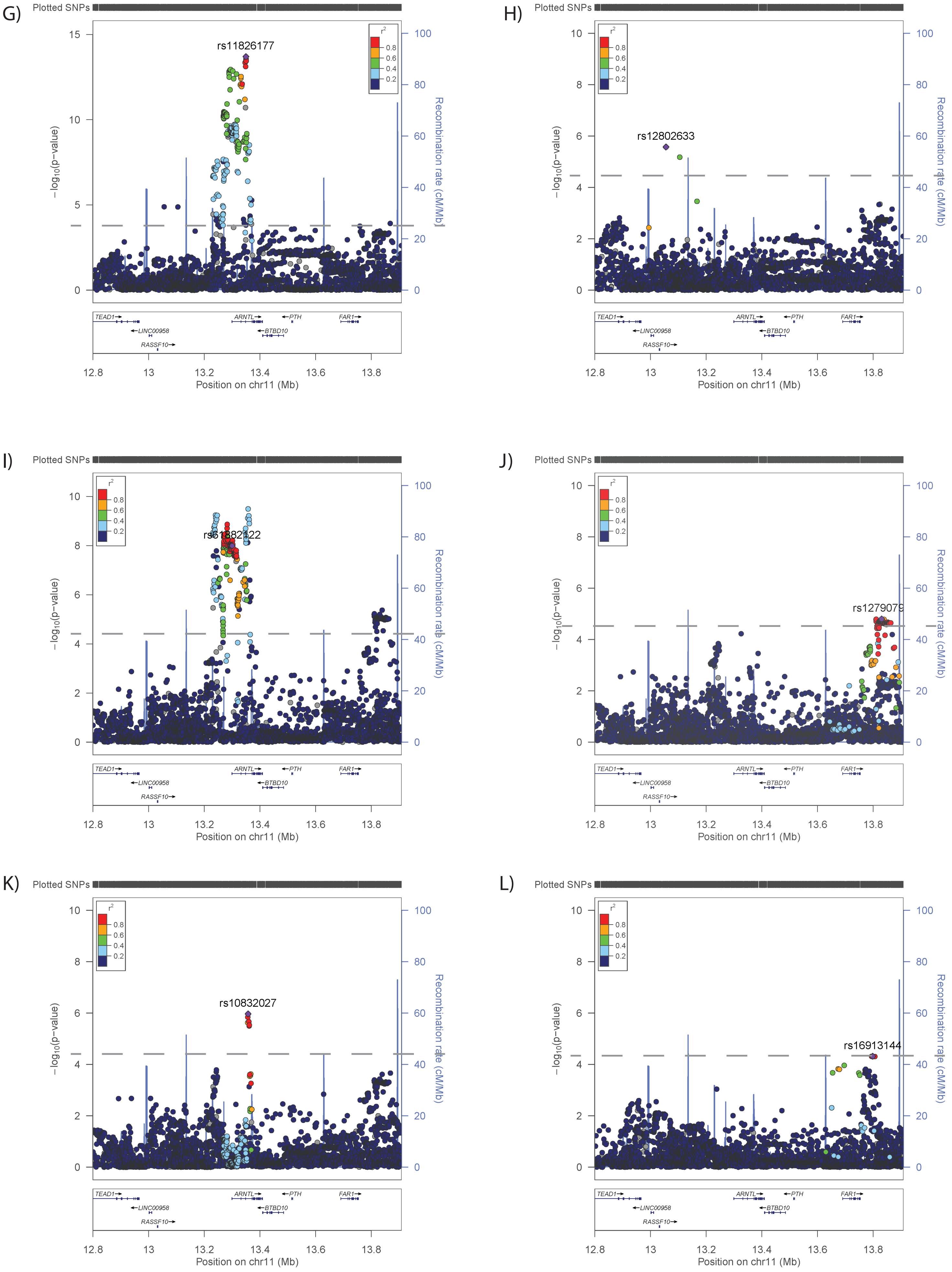
G)
Plotted SNPs
rs11826177
−log10(p−value)
Recombination rate (cM/Mb)
Position on chr11 (Mb)
H)
rs12802633
I)
rs61882122
J)
rs1279079
K)
rs10832027
L)
rs16913144
r2
0.8
0.6
0.4
0.2
TEAD1
LINC00958
RASSF10
ARNTL
BTBD10
PTH
FAR1
12.8
13
13.2
13.4
13.6
13.8

White British individuals

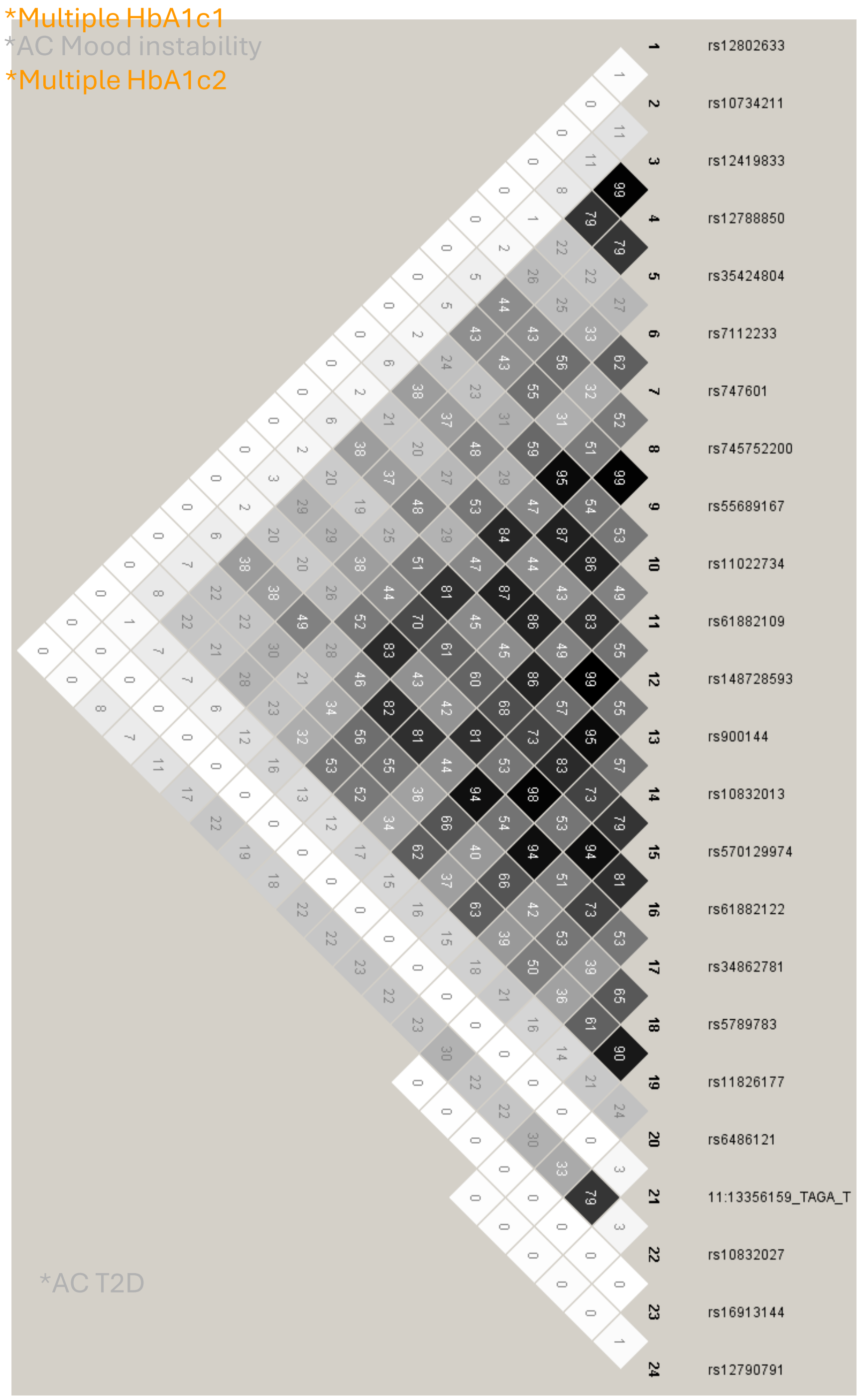

BMI2

Meta T2D

Risk-taking

Meta Risk-taking

Meta BMI 0

wEur DBPadj

Meta WHR

Neuroticism Meta Neuroticism

Meta Neuroticism 0

Multiple WHR

Meta SBPadj

Meta DBPadj 0

SBPadj

DBPadj Meta DBPadj

WHR1

WHR1 proxy

Anhedonia

Ever smoking Meta SBPadj 0

BMI1

Meta WHR 0

WHR2

WHR3

HbA1c

WHR2 proxy

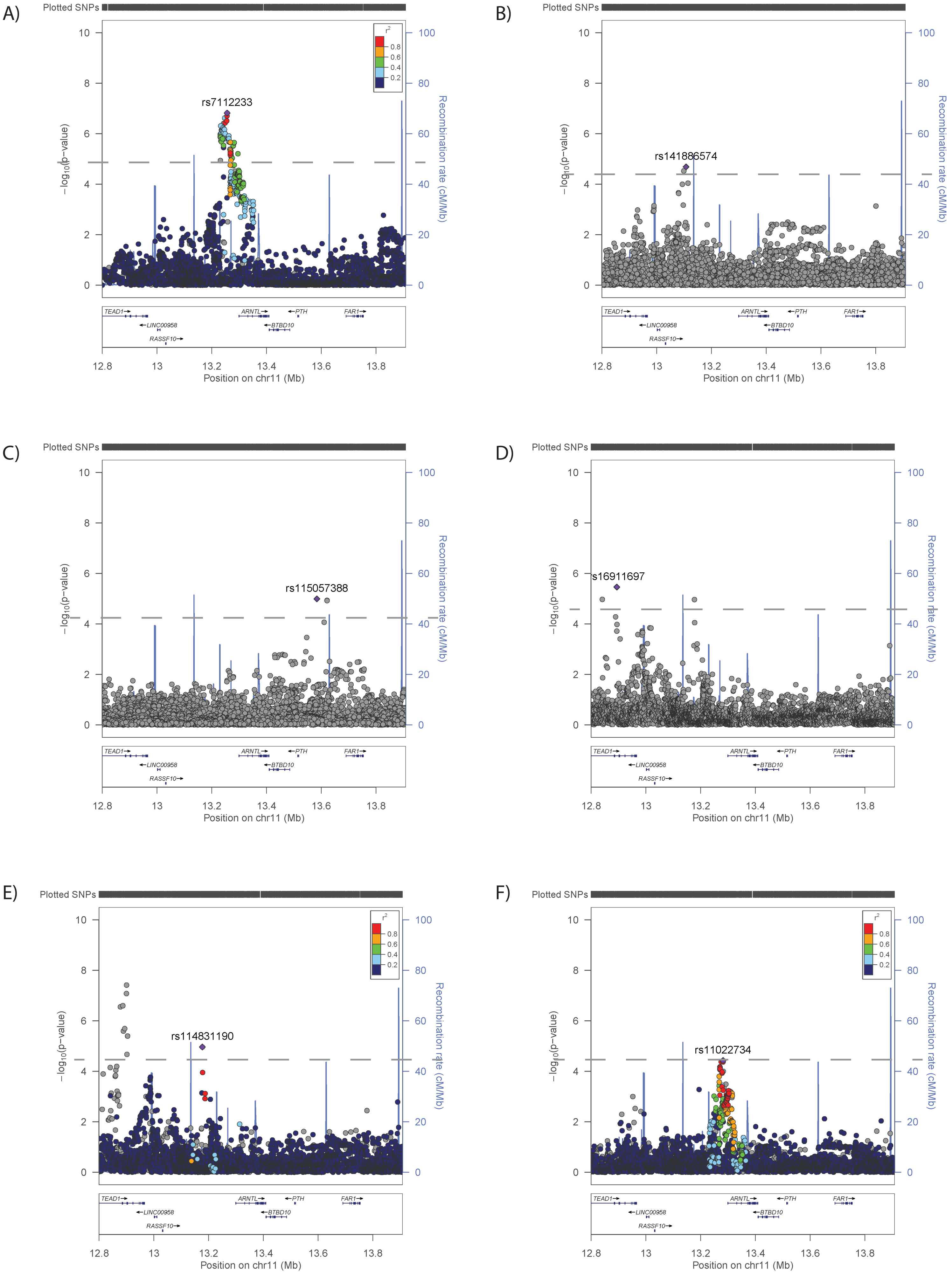
A)
Plotted SNPs
rs7112233
r2
0.8
0.6
0.4
0.2
B)
rs141886574
C)
rs115057388
D)
s16911697
E)
rs114831190
F)
rs11022734
−log10(p−value)
Recombination rate (cM/Mb)
TEAD1→
←LINC00958
RASSF10→
ARNTL→
←BTBD10
←PTH
FAR1→
Position on chr11 (Mb)
12.8
13
13.2
13.4
13.6
13.8

White European individuals

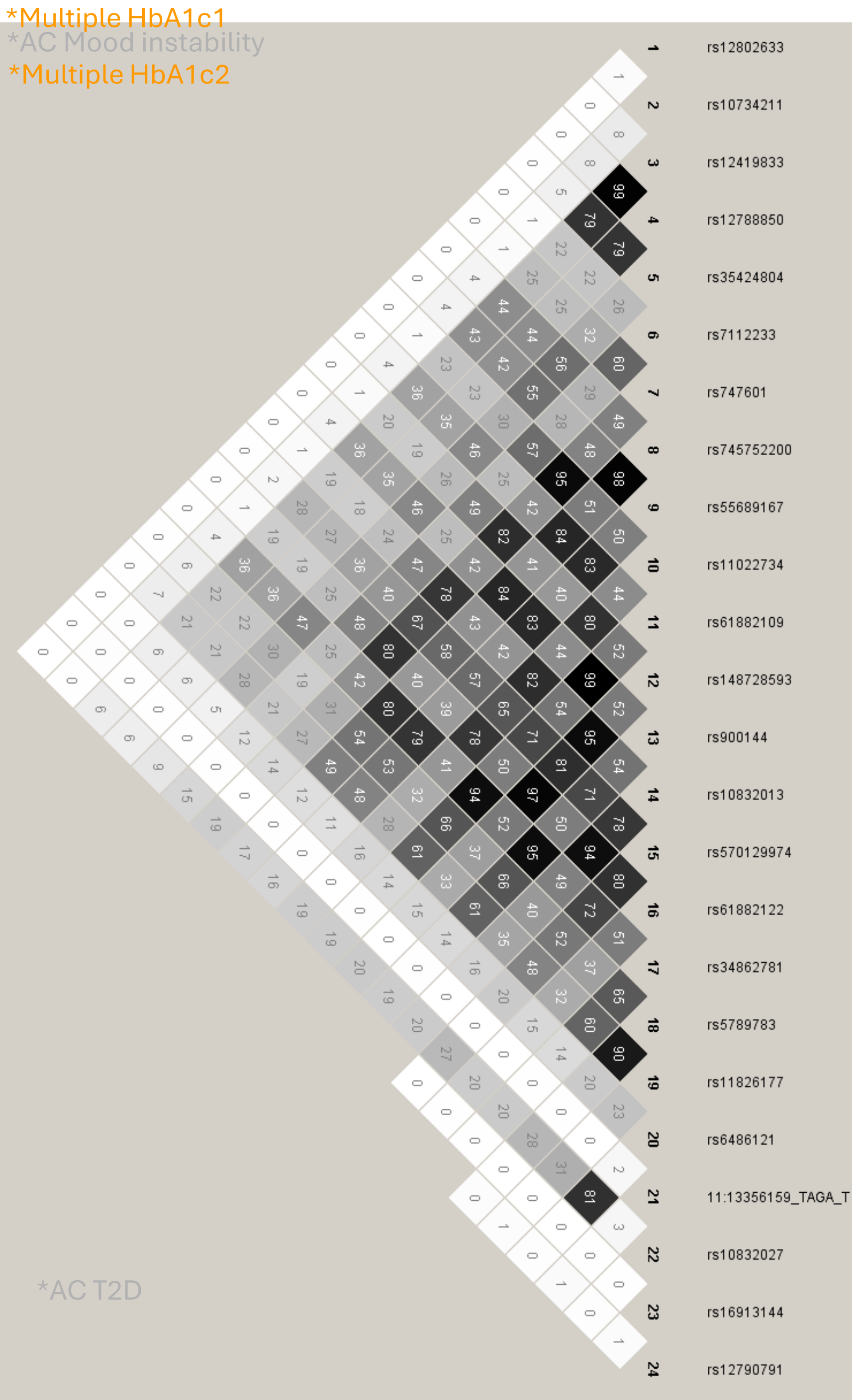

BMI2

Meta T2D

Risk-taking

Meta Risk-taking

Meta BMI 0

wEur DBPadj

Meta WHR

Neuroticism Meta Neuroticism

Meta Neuroticism 0

Multiple WHR

Meta SBPadj

Meta DBPadj 0

SBPadj

DBPadj Meta DBPadj

WHR1

WHR1 proxy

Anhedonia

Ever smoking Meta SBPadj 0

BMI1

Meta WHR 0

WHR2

WHR3

HbA1c

WHR2 proxy

South Asian individuals

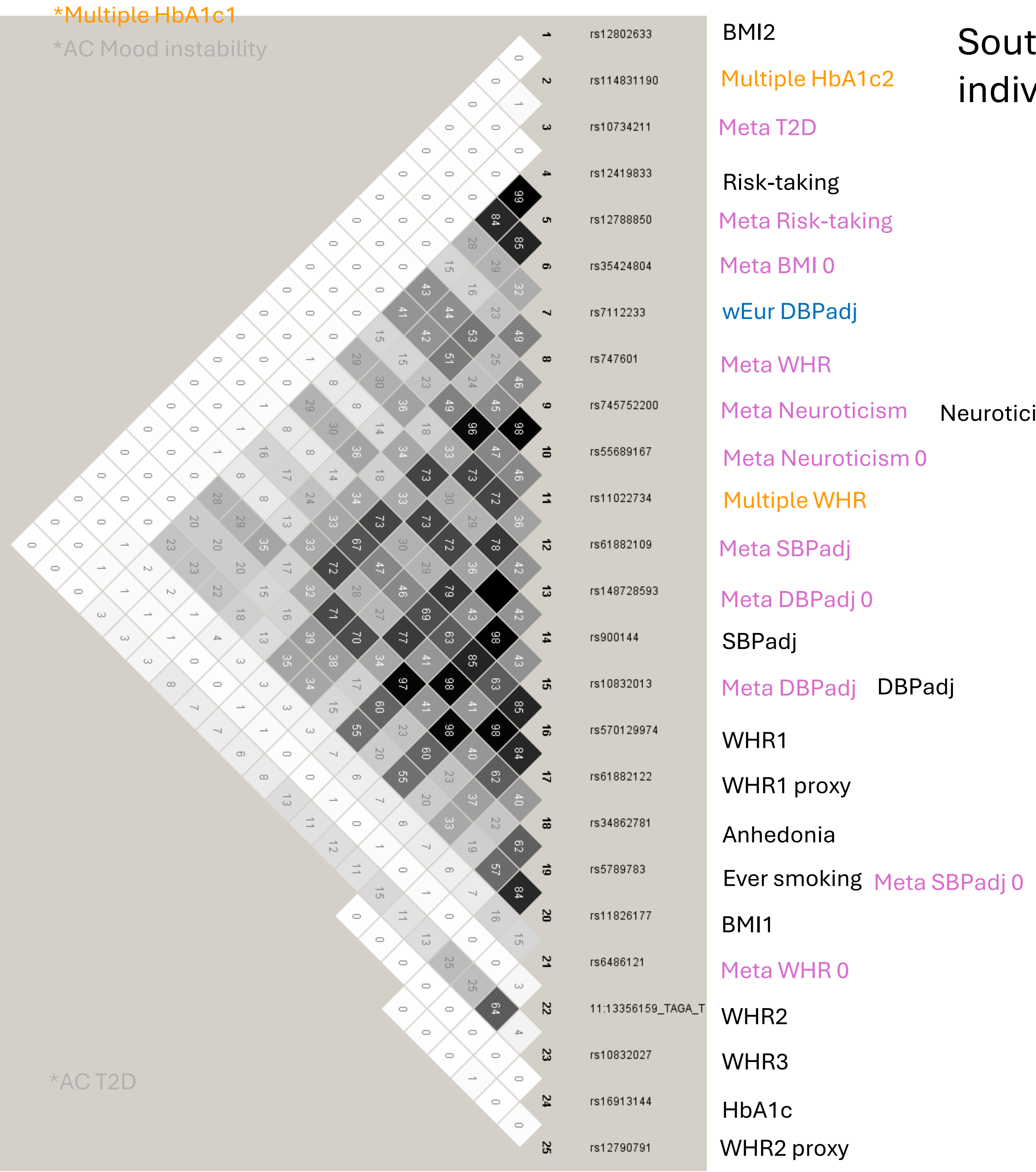

African Caribbean individuals

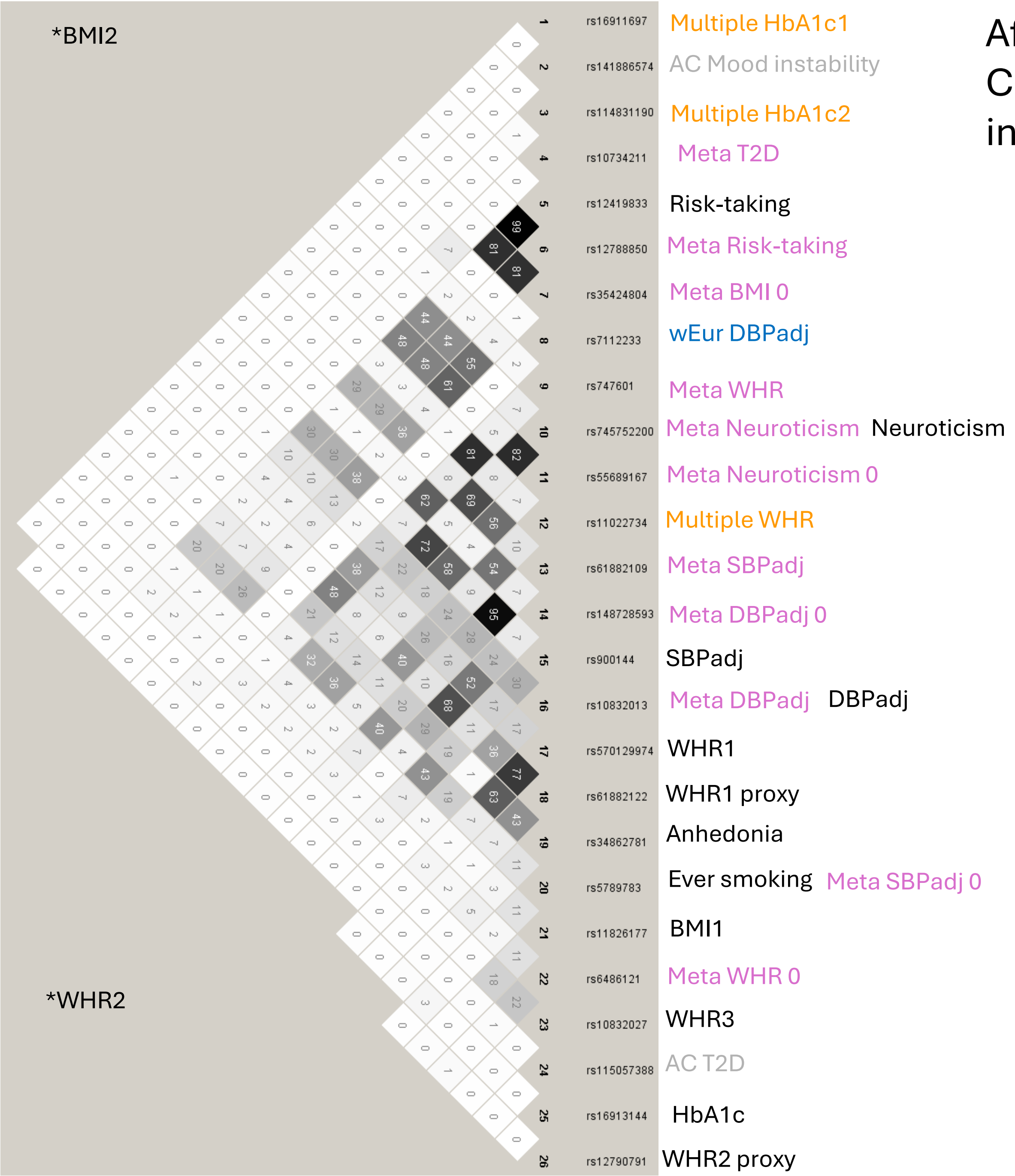

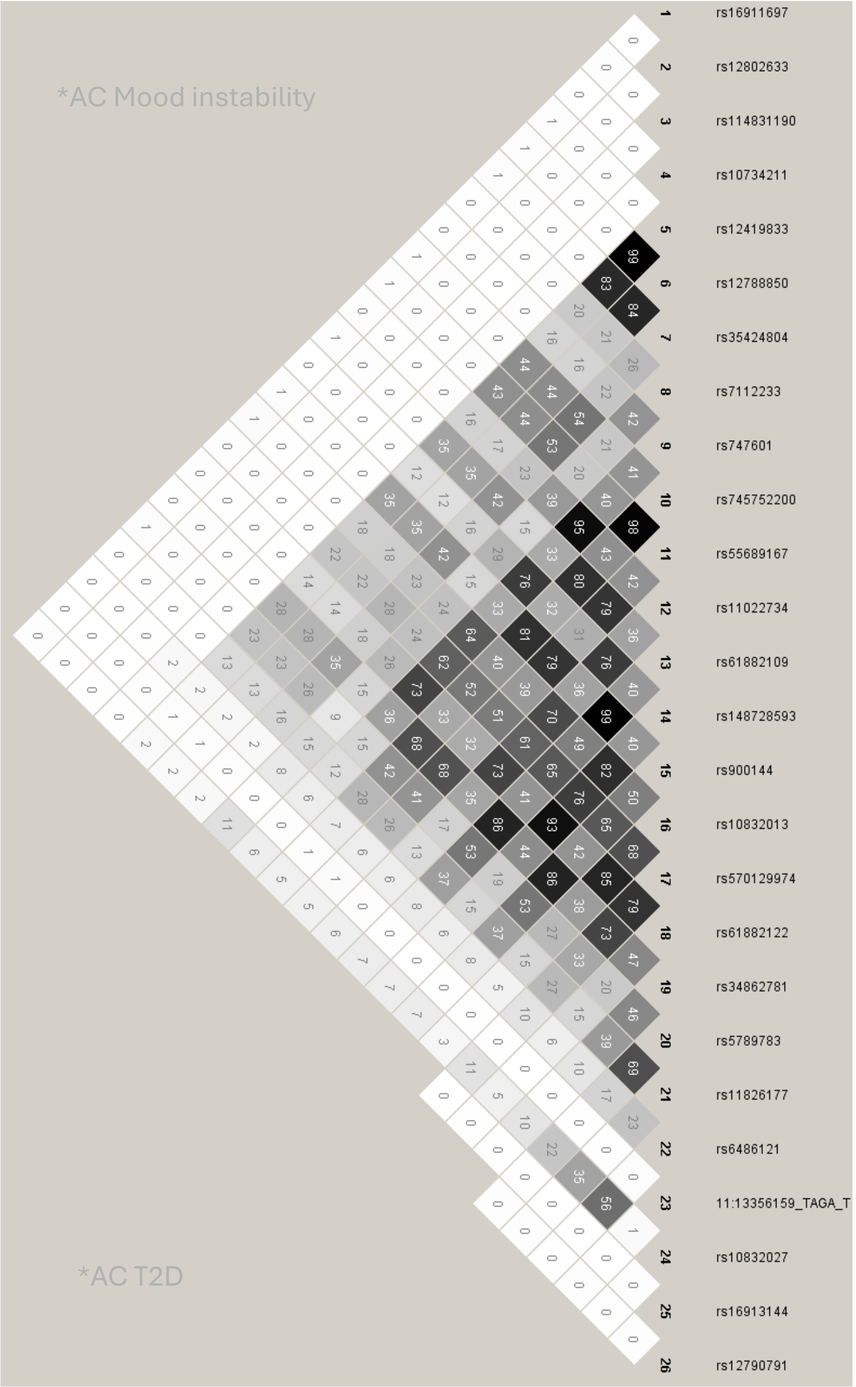

Multiple HbA1c1
BMI2
Multiple HbA1c2
Meta T2D
Risk-taking
Meta Risk-taking
Meta BMI 0
wEur DBPadj
Meta WHR
Neuroticism Meta Neuroticism
Meta Neuroticism 0
Multiple WHR
Meta SBPadj
Meta DBPadj 0
SBPadj
DBPadj Meta DBPadj
WHR1
WHR1 proxy
Anhedonia
Ever smoking Meta SBPadj 0
BMI1
Meta WHR 0
WHR2
WHR3
HbA1c
WHR2 proxy

Multiple Ancestries individuals

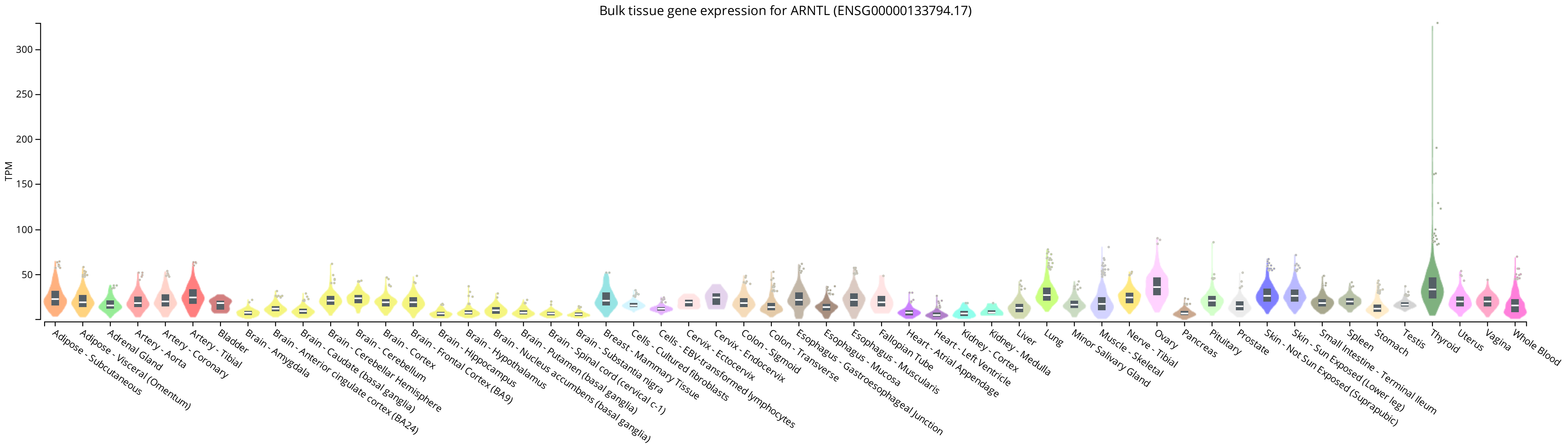
Bulk tissue gene expression for ARNTL (ENSG00000133794.17)
TPM
300
250
200
150
100
50
Adipose - Subcutaneous
Adipose - Visceral (Omentum)
Adrenal Gland
Artery - Aorta
Artery - Coronary
Artery - Tibial
Bladder
Brain - Amygdala
Brain - Anterior cingulate cortex (BA24)
Brain - Caudate (basal ganglia)
Brain - Cerebellar Hemisphere
Brain - Cerebellum
Brain - Cortex
Brain - Frontal Cortex (BA9)
Brain - Hippocampus
Brain - Hypothalamus
Brain - Nucleus accumbens (basal ganglia)
Brain - Putamen (basal ganglia)
Brain - Spinal cord (cervical c-1)
Brain - Substantia nigra
Breast - Mammary Tissue
Cells - Cultured fibroblasts
Cells - EBV-transformed lymphocytes
Cervix - Ectocervix
Cervix - Endocervix
Colon - Sigmoid
Colon - Transverse
Esophagus - Gastroesophageal Junction
Esophagus - Mucosa
Esophagus - Muscularis
Fallopian Tube
Heart - Atrial Appendage
Heart - Left Ventricle
Kidney - Cortex
Kidney - Medulla
Liver
Lung
Minor Salivary Gland
Muscle - Skeletal
Nerve - Tibial
Ovary
Pancreas
Pituitary
Prostate
Skin - Not Sun Exposed (Suprapubic)
Skin - Sun Exposed (Lower leg)
Small Intestine - Terminal Ileum
Spleen
Stomach
Testis
Thyroid
Uterus
Vagina
Whole Blood
